# The potential of Gantry beamline large momentum acceptance for real time tumour tracking in pencil beam scanning proton therapy

**DOI:** 10.1038/s41598-020-71821-1

**Published:** 2020-09-18

**Authors:** Giovanni Fattori, Ye Zhang, David Meer, Damien Charles Weber, Antony John Lomax, Sairos Safai

**Affiliations:** 1 Center for Proton Therapy, WMSA/C14, Paul Scherrer Institute, 5232 Villigen, Switzerland; 2 Department of Radiation Oncology, University Hospital Zurich, 8091 Zurich, Switzerland; 3 Department of Radiation Oncology, University Hospital Bern, 3000 Bern, Switzerland; 4 Department of Physics, ETH Zurich, 8092 Zurich, Switzerland

**Keywords:** Particle physics, Cancer, Biomedical engineering

## Abstract

Tumour tracking is an advanced radiotherapy technique for precise treatment of tumours subject to organ motion. In this work, we addressed crucial aspects of dose delivery for its realisation in pencil beam scanning proton therapy, exploring the momentum acceptance and global achromaticity of a Gantry beamline to perform continuous energy regulation with a standard upstream degrader. This novel approach is validated on simulation data from three geometric phantoms of increasing complexity and one liver cancer patient using 4D dose calculations. Results from a standard high-to-low beamline ramping scheme were compared to alternative energy meandering schemes including combinations with rescanning. Target coverage and dose conformity were generally well recovered with tumour tracking even though for particularly small targets, large variations are reported for the different approaches. Meandering in energy while rescanning has a positive impact on target homogeneity and similarly, hot spots outside the targets are mitigated with a relatively fast convergence rate for most tracking scenarios, halving the volume of hot spots after as little as 3 rescans. This work investigates the yet unexplored potential of having a large momentum acceptance in medical beam line, and provides an alternative to take tumour tracking with particle therapy closer to clinical translation.

## Introduction

In radiotherapy, the most intuitive and efficient way to treat a tumour that moves as a result of patient breathing is to track its position in real time with the beam, thus keeping minimal safety margins and maximising beam-on time. However, this so-called tumour tracking technique is usually referred to as “challenging”^1^ or “unlikely to be implemented clinically anytime soon”^2^ in particle radiotherapy, partly due to the difficulty to assess the tumour motion reliably enough and in real time^1,3^. Recent advances in image guided radiotherapy and clinical proton facility design have introduced on-line radiographic imaging during treatment and/or continuous monitoring of the patient surface^4,5^, not to mention future directions of magnetic resonance guided therapy^6,7^. As such, the main challenge now is how to adapt the treatment on-line following the patient anatomy, and how to perform this in a reliable and flexible way. In this study, we explore the potential of a novel approach to tumour tracking with an eye on the performance of the future generation of proton gantries, aiming at real-time treatment adaptation with the highest beam quality for clinical applications.

Tumour tracking has been implemented in photon therapy for a number of years^8–12^, even if it is still a relatively rarely used approach. However, the dose deposited by photons decays exponentially with depth as it traverses through the body, and therefore real-time tracking can be effectively implemented by laterally shifting the geometrical isocenter of the therapeutic beam to follow the tumour target. This functionality has been implemented in commercial photon machines by either moving the entire linear accelerator with a robotic arm, (e.g. Accuray CyberKnife) or with a gimbaled MV LINAC with a multi leaf collimator (MLC) (e.g. Brainlab ExacTrac VERO^13^). Other options available, albeit at an experimental stage, are dynamic MLC tracking^8,14^ and real-time compensation with couch motions^15,16^. Extensive use of online image guidance is made to monitor the target motion during the treatment, using on-board X-ray imaging to either track directly the tumour and related structures or, more often, fiducial markers implanted in its close proximity. Non-ionising alternatives are commercially available and rely on correlation models to estimate the target position from the body surface motion or ultrasound imaging. For a thorough review of the use of imaging for tumour tracking the reader is referred to Riboldi et al.^3^. More recently though, magnetic resonance imaging has been integrated in radiotherapy linear accelerators from commercial vendors, opening up new possibilities for on-line soft tissue imaging in the context of advanced radiotherapy workflows and motion mitigation^17^. The translation of these techniques to particle therapy is challenging, and presents a number of very specific issues dictated by the finite range of the treatment beam and its dependence on the density of tissues crossed. Successful tumour tracking with particles therefore is strictly interrelated with accurate modelling of the whole patient anatomy in the beam path^18^ and the rapid, on the fly, adaptation of beam settings to avoid overshooting into healthy tissues or severe distortions of the dose in the target^19^. This latter aspect, related to on-line control of dose delivery to track intra-fractional organ motion is the subject of this contribution, with specific reference to proton treatments delivery with pencil beam scanning (PBS).

During the pencil beam scanning delivery process, spot (Bragg peak) positions are set by the therapy control system (TCS) individually. Although transversal beam position changes can be performed quickly, allowing for fast lateral tracking of the tumour, modulation of energy, in order to also track the tumour in depth, is problematic due to the typically long times required for energy layer switching on most gantry systems. However, this is not due to the time it requires to change the position of the energy degrader or resetting the energy selection system, but more due to the time it takes to change the magnetic field of the dipoles magnets in order to preserve the position of the pencil beam at isocentre. For instance, in PBS treatments, the beamline magnets are repeatedly set, or technically speaking “tuned”, to follow the sequence of energies in the plan. The beam momentum variation between subsequent energy levels is about 1% and its application takes from a hundred milliseconds or so on fast machines like PSI Gantry 2, to more than 1 s in some facilities. Moreover, beamline magnetic ramping is generally performed only in one direction, e.g. from the highest to the lowest energy, to avoid complex hysteresis effects in the magnets^20^. As a result, treatments are delivered as a sequence of discrete iso-energy layers while scanning transversally through the target with limited capacity for fast energy modulation^21^.

The most sophisticated prototype for beam tracking experiments with particle beams was developed at the fixed beamline for carbon ions in the GSI Helmholtzzentrum für Schwerionenforschung^22–24^. In the GSI setup, a wedge-based beam degrader installed downstream of the beam line nozzle was used to achieve real time energy modulation, while the beam was offset laterally by adapting the scanning magnet settings. Downstream degradation however, particularly for protons, can substantially broaden the pencil beam size, potentially compromising the lateral fall-off and dose penumbra. In addition, such an approach would require a major upgrade to existing proton therapy facilities.

Recent results on gantry design research inspired us however to rethink the energy modulation approach towards a more elegant solution that does not require a dedicated degrader downstream in the beamline and is therefore potentially applicable for most clinical units. For instance, aiming for reduction of costs and size for particle therapy facilities, PSI researchers recently proposed a compact gantry design using superconducting magnets^25,26^. This technology is capable of a large momentum acceptance (over ± 10%) so that the beam energies required for patient treatments can be adjusted up-stream, without re-tuning the entire beamline^27,28^. The globally achromatic design of existing gantries ensures that particles with energy within a defined momentum acceptance can be transported without chromatic dispersion. In other words, even particles which have non-nominal momentum will be focused in the same point at iso-center after transport through the gantry. As such, ultra-fast and continuous energy regulation within the momentum acceptance can be realised with a mechanical degrader or energy selection system located upstream of the gantry, without the latency overhead for re-tuning the entire beamline. This provides a unique opportunity to realise tumour tracking with clinically acceptable beam shape and beam monitoring systems, while the gantry achromaticity ensures accuracy in beam positioning in the patient.

**Figure 1:**
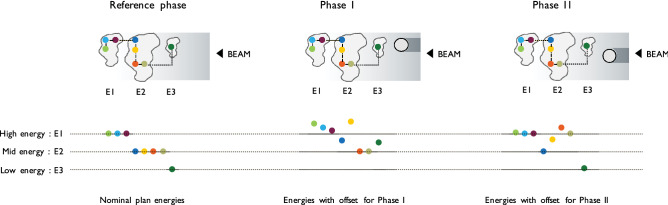
Sketch of energy offsets for beam tracking in a simplified treatment plan consisting of 8 spots distributed over three energy levels (E1, E2, E3). Reference spot energy is altered as a function of a high-density structure (e.g. rib cage bones) crossing the field during patient breathing. Individual spot energy is increased as function of material density in his path. Three motion phases are displayed (Reference, Phase I, Phase II).

Planning for tumour tracking starts with the acquisition of time resolved 4D imaging^29^ such as 4DCT or 4DMRI. A treatment plan is then typically optimised on one selected reference image, and the remaining phases used to derive lateral position offsets and energy changes required to track each Bragg peak (also referred to as a ‘spot’), by taking into account the tumour position and any deforming anatomies through which the beam needs to pass (Fig. 1). The momentum acceptance of clinically available beamlines is however reduced compared to aforementioned studies on superconductivity and therefore tracking corrections cannot be selected arbitrarily on-the-fly without frequent beamline tune changes. Our strategy for making use of even a relatively small acceptance is to drop the canonical scanning path in favour of a less regularised meandering scheme, which follows the deforming anatomy of the patient. By making reasonable assumptions about the breathing pattern reproducibility, it is possible to generate a different type of spot list that is not structured in a sequence of iso-energy layers scanned across with spots on a regular grid, but that instead takes into account the progressive anatomical changes. In a respiratory correlated irradiation, the patient breathing and the beamline ramping run in synchrony and the spots that, including tracking offsets, match the instantaneous combination of breathing phase and available energy are progressively selected for delivery. Leveraging on the flexibility of PBS units in setting the transversal beam position, the monotonic progression of energies is the driving criteria of such a meandering scheme that requires fewer beamline tunes, but instead, a continuous energy regulation within each tune’s respective momentum acceptance. In addition, energy meandering was introduced to eliminate the dead time required for beam line ramping, for an optimal treatment duty cycle. In the absence of specific 4D planning optimisation, inhomogeneities may be expected in the entrance channel, upstream to the target and in the target itself^30^. This problem is further addressed by combining tumour tracking with rescanning in order to mitigate the dose corruption through multiple deliveries of the plan under different interplay conditions.

The feasibility of ultra-fast energy changes within the beam line momentum acceptance, exploiting the high level of achromaticity of next generation treatment gantries, opens new and unexplored opportunities for beam tracking applications and on-the-fly treatment adaptation. In this paper therefore, we investigate the potential of this approach through simulations using first simple geometric cases in order to fully understand the advantages and limitations of the approach in easy to interpret geometries, and then in an example clinical case.

## Results

Tumour tracking results are reported for single field, single fraction treatment of four test cases (Table 1) using 4D dose calculation^18^.

**Table 1:** Dataset characteristics including PTV/CTV size, motion amplitude and period, volume deformation observed over 4D imaging.

	Description	PTV size [cm^3^]*	CTV size [cm^3^]*	Motion amplitude, PTV center of mass [mm]	Motion period [s]	PTV volume deformation [%]
S1	Sphere 8 cm diameter	260.672	227.86	SI: 20	3	0
S2	Sphere 4 cm diameter	31.636	23.908	SI: 20	3	0
S3	Sphere 2 cm diameter	3.724	2.012	SI: 20	3	0
P1	Liver cancer patient	365.353	314.59	LL: 2.18; AP: 2.18; SI: 18.63	7.22	+ 2.95/− 6.57

*Actual values from the contours drawn on sub-sampled image volumes used for treatment planning.

Three geometric moving phantoms S1, S2 and S3 simulate increasingly smaller spherical targets moving behind a dense cylindrical rod. The structures’ densities have been set to mimic the treatment of soft tissue tumours (100 HU) moving in a water bath (0 HU), behind a bony element of the rib cage (1,000 HU). Simplified rigid motion of 20 mm range, transversal with respect to the beam, has been considered for these cases and sampled in 10 synthetic CT phases. P1 is a liver cancer patient published in Zhang et al.^31^ (Patient III, Motion B) with a medium size tumour in the anterior superior segment (VIII) of the right lobe. The 4D CT images used in this study were obtained by warping one phase of the patient 4DCT with the deformation fields derived from 4D MR imaging to generate 20 motion phases with temporal resolution of 2.7 Hz^32^. Patient breathing has been modelled using a multiresolution B-spline deformable image registration algorithm implemented in the Plastimatch software^33^. With this approach, clinically plausible breathing irregularities can also be modelled to more thoroughly test proposed 3D tracking approach. In these data sets, the tumour moves 2 cm primarily in the superior-inferior direction during breathing, as well as deforming up to about 10% of its volume. In all cases, a planning target volume (PTV) has been obtained using a 2 mm isotropic expansion of gross tumour volume, and planned with a prescribed dose of 1 GyRBE. The PTV margin has been kept intentionally small, much lower than clinically used expansions, such as to make the plans sensitive even to the smallest dose disturbances that would potentially be hidden by the use of larger safety margins instead. With this choice, adequate treatment conformity (V95 > 95%) was ensured in static conditions for all cases but S3, in which the small size of CTV and synthetic bone structure compared to the beam size at target depth limit the dose conformity. Nevertheless, to ensure consistency in our plan comparisons, S3 has also been planned with 2 mm margins. All plans consisted of a single field resulting from Single Field Uniform Dose (SFUD) optimisation. Gantry momentum acceptance considered was $$  {\raise0.7ex\hbox{${\Delta p}$} \!\mathord{\left/ {\vphantom {{\Delta p} p}}\right.\kern-\nulldelimiterspace} \!\lower0.7ex\hbox{$p$}} =  \pm 0.6\%    $$, a realistic value for PSI Gantry 2 and commercial treatment units^34^.

The International Commission on Radiation Units and Measurements (ICRU) recommendations were followed to evaluate dose quality for conformity $CI:=\frac{{TV}_{{D}_{98\%}}}{PTV}$ , defined as ratio between the volume of the 98% isodose and the PTV, and hot-spots (HS) as the volume of tissue outside the 2 mm expansion of PTV that receives a dose greater than the target prescription. In addition, CTV dose coverage and homogeneity have been evaluated as V_95%_ (the volume of the target receiving at least 95% of the prescription dose) and the homogeneity index defined as $HI:={D}_{5\%}{-}{D}_{95\%}$.

**Figure 2:**
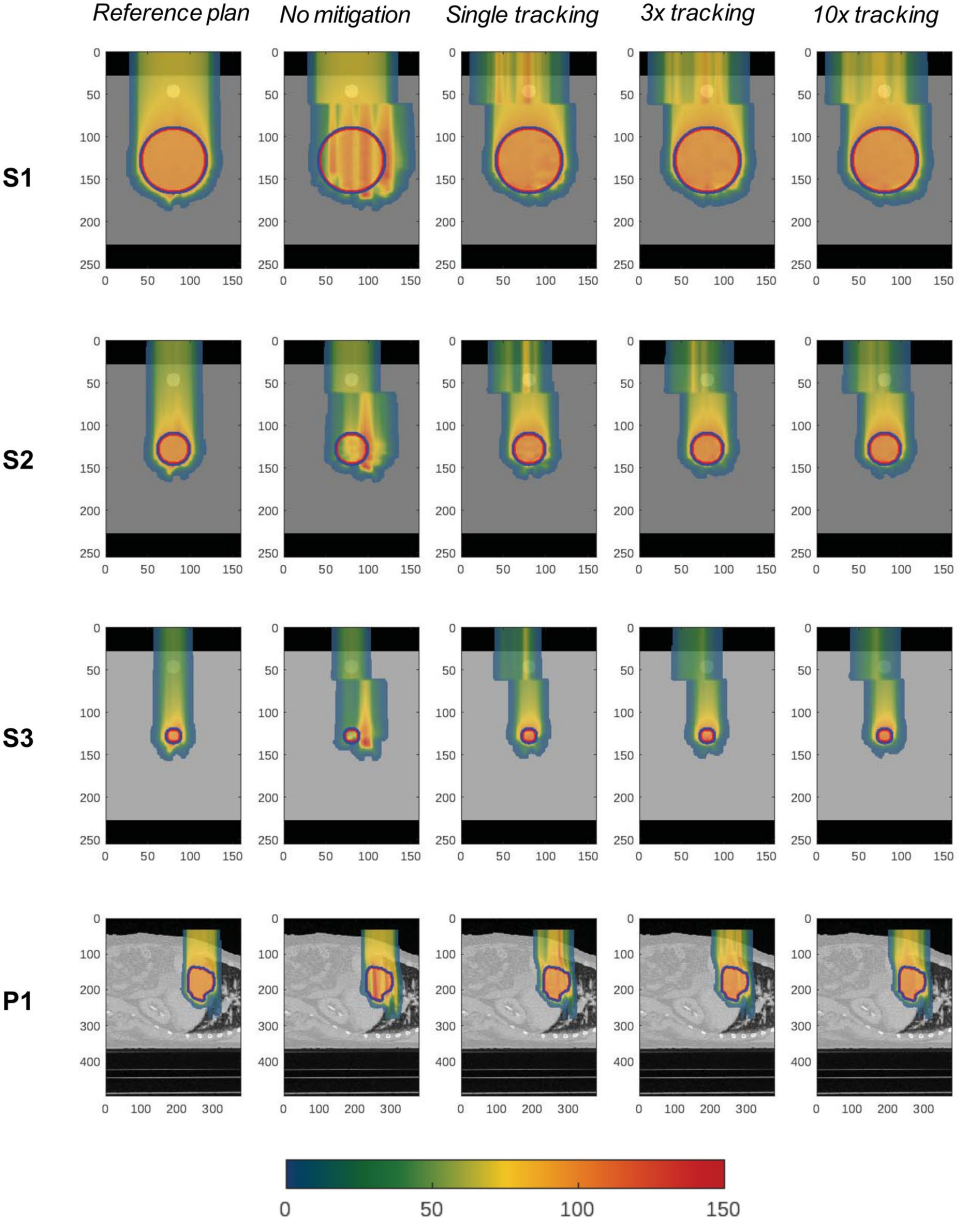
Dose distributions on sagittal plane cut at isocenter. Reference plan delivered on stationary anatomy is compared with 4D dose calculation without motion mitigation and for three rescan factors. PTV and CTV contours are shown respectively in blue and red. Axes are millimetres, dose expressed as percent of CTV prescription.

Dose quality indices for single tracking deliveries under the condition of motion (Fig. 2) were compared with reference values in stationary condition and free-breathing treatments without mitigation (Table 2). Four *tracking scenarios* have been simulated and compared: (1) with (WEM) and (2) without (WoEM) energy meandering, and (3) starting at high or (4) low energies. Treatment conformity is recovered exceptionally by means of tumour tracking for all cases but S3, where despite a significant improvement on the median value (− 85% of unmitigated case) a large range of variation is reported among the different tracking techniques. Inhomogeneity is within 20% for S1 and S2, with larger median values for S3 and P1 and substantial variations among scenarios for the former. Single tracking is also shown to restore V95 for all geometric phantoms, whereas the liver cancer case scores a sub-optimal 76.9%. Similarly, the volume of hot spots is marginal in S1, S2 and S3 but are comparable to the unmitigated case for the patient data simulations.

**Table 2:** Median (range) of dose quality indices of four different single tracking modalities.

	CI			HI [%]			V95 [%]			HS [cm^3^]		
S1	4.1	1.5 (0.3)	1	65.3	10.4 (0.7)	3.1	49.9	94.9 (3.1)	99.3	26.06	1.3 (3.1)	0.18
S2	9.3	2.3 (0.2)	1.3	73.4	18.4 (4.8)	5.4	23.6	92.4 (8.4)	97.4	6.72	0.1 (0.3)	0.12
S3	43	6.4 (10.4)	2.1	42.7	33.1 (26.3)	12.9	0	91.5 (15.4)	86.8	1.95	0.3 (0.6)	0.06
P1	3.8	1.5 (0.1)	1.1	65.3	24.9 (5.9)	5.5	48	76.9 (5.7)	98.6	20.03	20.6 (11.1)	1.67
	No mitigation	Tracking	Stationary	No mitigation	Tracking	Stationary	No mitigation	Tracking	Stationary	No mitigation	Tracking	Stationary

Reference values in stationary and unmitigated free-breathing conditions are reported for comparison.

The delivery times for each simulated scenario have been estimated using the timing model of PSI Gantry2 from Klimpki et al.^35^ (Table 3). The model is built on machine log files from patient treatments and parametrises the energy dependency of beam current transmission between 70 and 230 MeV. Relevant for this study are the mean time required to initialise all beam line elements (full ramping) and mean dead time for small energy changes of clinical treatments, which are respectively set at 9.3 s and 106 ms. Additionally, the model has been extended to simulate energy meandering, in which the beamline is only transitorily set at the corresponding energy bound, obviating the need of a full ramping^20^. In this case, the dead time on our machine is 1 s. Based on this, the duration of tracking treatments using energy meandering was only marginally higher than the conventional plan without tracking. Without meandering in energy, several full beam line rampings during the delivery would substantially increase the treatment time, which is at least double the duration of conventional plans. The selection of starting energy, whether high at 230 MeV or low at 70 MeV was shown to have only a minor role in most cases.

**Table 3:** Delivery time of tracking plans with (WEM) and without (WoEM) energy meandering considering the starting energy dependency (High or Low).

	Delivery time [s]				
	Standard	Tracking WEM		Tracking WoEM	
S1	41.5	49.7	53.3	74.2	87.2
S2	9.9	16.2	16.2	31.1	30.8
S3	3.3	4.9	5.6	13.2	25.8
P1	47.3	58.3	57.9	98.7	91.1
Starting Energy	High	High	Low	High	Low

Reference values for conventional deliveries without tracking are reported as ‘Standard’.

**Figure 3:**
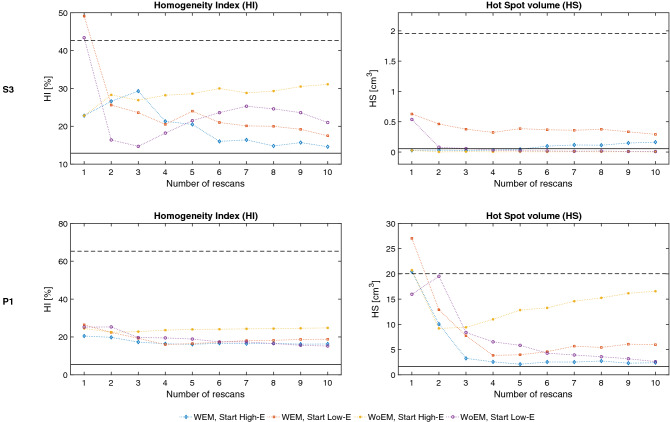
CTV homogeneity (HI) and hot spots volume (HS) outside the PTV as a function of the rescan factor, for each tracking modality; with (WEM) or without (WoEM) energy meandering and starting with Low or High energy. Results from stationary irradiation and 4D dose calculation without tracking are shown respectively as solid and dashed black lines.

The small sphere target (S3) and liver patient (P1) are challenging cases to treat with proton beams. The 2 cm diameter sphere S3 is rather small compared to the proton beam size at the target depth, impairing the plan quality even in stationary conditions. Moreover, during the breathing cycle, it moves completely out of the shade of the dense cylindrical rib model, requiring substantial energy adaptation. P1 has a tumour in the upper lobe of the liver whose apex is neighboured proximally and distally by air in the right lung, a steep density gradient to account for in proton treatments. Implanted metallic seeds and the volume deformation impact further on the complexity of P1 test set. For those cases therefore, we have analysed in more detail the effect of each tracking technique and the potential of rescanning to reduce target inhomogeneity and hot spot in proximal tissues upstream of the target (Fig. 3).

Meandering in energy when rescanning has a positive impact on target homogeneity, resulting in more stable HI indices approaching the values of the stationary condition. This effect is pronounced on the spherical test case S3, whereas in the liver patient P1 its impact is hidden by the fact that the simple Bragg peak tracking technique without specific 4D plan optimisation is reaching its limits of homogeneity. Differences between the starting energy directions can be observed for S3, even though this is not significant considering the full cohort. Hot spot analysis is more appropriate for the patient data, as the homogeneous media in which the sphere target is moving is more forgiving for hot spots. Similarly, hot spots outside the targets in P1 are mitigated by rescanning with a relatively fast convergence rate for most of tracking modalities, halving the HS volume after as little as 3 rescans. In this regard, the *tracking scenario* simulated is not a significant parameter, except for the case of rescanning starting from low energies and without meandering, that diverges with the increase of the number of rescans.

## Discussion

Elaborating on tumour tracking is an opportunity to investigate the yet unexplored potential of exploiting large momentum acceptance of proton gantries and beam lines to achieve ultra-fast energy regulation and beam offsets. In fact, we build on the latest available results regarding the delivery of tumour tracking treatments in particle therapy to address the energy regulation from a different perspective, thus providing a solution that does not require significant changes in the beamline and capable of clinical level beam quality. As demonstrated in this proof-of-concept study, the method is indeed applicable to clinical cases, with dosimetric results that are well aligned with the reference literature on tumour tracking. Unlike all currently available implementations however, potentially our concept does not require any additional hardware and this may be the key for its translation into the clinic of facilities that feature an energy degrader upstream in the beamline, as most of the running treatment units are.

### Fast energy regulation

Magnetic beam deflection on a static wedge degrader as described by Chaudhri et al.^36^ is possibly the fastest method to control the energy, promising beam energy selection on the same time scale as lateral scanning. This is however not the standard of clinical units that, most often, rely on motor driven double-wedge^37^ or multi-plates^38^ mechanical degraders. Albeit having lower performance, such mechanical devices have already been used to regulate the energy on a spot-by-spot basis in experimental tumour tracking studies^24^. Adopting time prediction to compensate for communication delays, around 20 ms actuation time for around 5 mm water equivalent range shift is achieved in those cases. The wedge travel to regulate the energy within the momentum acceptance is however expected to be a fraction of that value, making it theoretically possible to reach fast performances. Moreover, our pencil beam scanning implementation is a discrete (step-and-shoot) method, i.e. the beam is turned off in between of the spots, with a dead time of 2.83 ms (mean)^35^ that leaves some margin for controlling the energy degrader.

### Beam losses

A realistic model of the dose delivery dynamic is essential in the process of spot sorting, to generate a similar delivery sequence that is synchronous with the patient breathing. Critical in this regard is the accuracy in the estimate of the current loss for off-momentum particles. Particles with non-nominal momentum within the acceptance, ± 0.6% by design in our case study, although transported through the gantry, may be chromatically dispersed in the beamline, leading to transmission losses. This should not however be problematic in current cyclotron-based facilities, reporting extremely low latency in current control (nominally 100 microseconds at PSI^34^) and therefore being compatible with tracking applications and on-the-fly current corrections. Besides, as the beam line transmission is a function of the particles momentum within the acceptance, the current corrections can be pre-computed at the time of treatment planning and made available for delivery. Accordingly, in this simulation study the beam current at isocentre for all energies within the beamline acceptance is therefore assumed to be in line with the clinical standard of Gantry 2 operation at PSI. Notwithstanding, this modality requires high standards in the regulation of the accelerator current such as to restore beam intensity at iso-center as planned.

### Tumour tracking with a limited momentum acceptance

Momentum acceptance is a well-defined property of the beamline, which depends on the design and configuration of elements, and whose width sets the boundaries of beam energy regulation. This may be less of an issue for next generation gantry designs but it can be limiting in existing facilities that have just a few percent acceptance. As has been shown in this study, however, even a limited band of ± 0.6% may have potential for beam tracking. For a 230 MeV proton beam, about 2 mm water equivalent range corrections can be set on the fly, without changing the beamline energy tune. This value scales with decreasing energies, and at the lower-end of clinical tunes (70 MeV) becomes so little to be meaningless. Therefore, to enable larger beam energy corrections, high energies should be preferred, adding an offset in the treatment plans if required. For this, a thick slab of compensating material close to the patient may be required to degrade from these higher energies to clinically relevant ranges (see e.g.^39,40^). Unlike the downstream wedge system used for experimental tracking measurements, such degrading material would be a fixed element that can be in close contact with the patient skin, thus minimising the beam broadening^39,41^.

### Energy meandering

The possibility to perform energy meandering is not only useful to improve the duty cycle of the treatment (see Table 3) but also provides additional flexibility in the spot sequence optimisation. Simply put, using the standard high-to-low energy progression would probably have a bias towards the use of pencil beams with high ranges and therefore favours the treatment of anatomical conditions where high density structures are in front of the target. The energy sequencing however can be flipped to low-to-high energy sorting to minimise the chances of treating through bones and complex heterogeneities. Whether to start with high or low energy, or introduce energy meandering, is however to be evaluated in accordance to the respiratory phase used to create the nominal plan. When treatment delivery starts from high energy in a breathing phase far apart of the planning image, high weight spots in the distal part of the target are delivered with systematically larger tracking offsets, thus increasing the probability of dose corruption and inverse interplay that are unlikely to be compensated throughout the treatment. An ideal case to observe this behaviour is S3, that indeed reports large variations in target dose conformity and homogeneity among the different tracking sequences. This effect is however mitigated by the application of rescanning, introducing random changes in the delivery sequences. Even very few rescans are enough to observe a significant improvement in the conformity and reduction of dose hot spots outside the target as reported in Fig. 3, for the small spherical target and clinical liver cancer patient.

### Tumour tracking and rescanning

As has been presented, our concept, which does not require re-optimisation of the original treatment plan, is prone to the inverse-interplay effect in tissues upstream of the target^18,23^. Target dose degradation can also be introduced by the pencil beam shape corruption due to the differences in tissue heterogeneities in the tracked geometry compared to the planning image^30^. The simplest approach to tackle such criticalities is rescanning—that averages out inhomogeneity over multiple rescans of the treatment field. Combining rescanning with our energy sequencing is however complicated. As we do not rely on canonical pencil beam meandering in iso-energy layers, the concept of layered rescanning^42^ does not fit with this implementation of tumour tracking. Volumetric rescanning^43^ instead has been integrated in the energy sequencing routine, by taking into account the time dependency of the delivery and avoiding repeated irradiation of spots in breathing phases that are close together. In our implementation, unlike conventional methods, the sequence in which the spots are delivered changes in each rescan, depending on the interplay between the respiratory phases and the progression of the beamline tunes. This, in fact, may bring additional stochasticity in the rescanning, with potential benefit on the overall effectiveness. It should be noted however, in our simulations, the minimum deliverable spot weight of our machine has not been considered explicitly. However, as shown in Fig. 3, for a clinically relevant case (P1) dose hot spots and homogeneity reported a rather quick convergence rate, reaching relatively stable values after just 2–3 rescans. In such a rescanning regime, minimum spot weight is not of concern, in particular considering that the beam current regulation in Gantry 2 is controlled on a spot-by-spot basis^44^.

Alternative approaches to compensate for inverse interplay can be found in the literature, addressing the problem at either the time of treatment planning like 4D optimisation^45^ or delivery, such as the case of real-time dose compensation from Lüchtenborg et al.^19^. These methods, however, are not the definitive solution to the problem of controlling energy in real time during dose delivery, but instead can take advantage of the method proposed here to take a step towards the implementation and testing in a clinical treatment unit, without the need for experimental hardware and instrumentation.

### Dealing with breathing irregularities and motion-induced density changes

In the most efficient possible realisation of tumour tracking, the patient, with his/her individual breathing pattern drives the treatment. We have instead described a breathing synchronised irradiation modality that is feasible taking into account technical and implementation constraints, but which however requires a certain prior knowledge of the breathing pattern. Treating irregular breathers would require however more freedom in energy regulation, not tied up to a strict ramping direction. Interestingly, this holds true within the energy bands, where both positive and negative range corrections are possible without changing the magnets settings. Therefore, should beamlines with large acceptances become reality, an option not considered in the present study, would provide improved flexibility for tumour tracking in presence of range variations. Moreover, the use of a spot list sorted by tracked energies as described would be the correct starting point, as range variations would occur more gradually compared to a normal beam meandering scheme that follows a sequence of energy layers.

Graeff^46^ has reviewed several approaches to 4D optimization that are of particular relevance for tumour tracking applications. By using the entire 4D image dataset during planning, these methods largely solve the drawbacks of simple 3D planning, which does not take into account the distortions in dose depositions along the pencil beam track and the interference pattern in the entrance channel (reverse interplay) resulting from tumour tracking. For the most part, these treatment techniques require the synchronization of the delivery with the respiratory movement and can therefore benefit from the method presented here, which is based on the same assumption but also can, in principle, take into account irregularities in breathing amplitude and base line shifts. The robustness aspects of 4D planning has recently been addressed by Wolf et al.^47^ using a dataset of lung cancer patients treated with rescanning, using worst-case optimisation to substantially improve the plan robustness against various treatment uncertainties, including range changes and deformable motions. However, at least for cases where the motion is too large to be mitigated with rescanning only or where the price to pay on the organs at risk to achieve target coverage with robust optimisation only would exceeds the clinical benefit, its combination with beam tracking should not be excluded.

Finally, the clinical realisation of tumour tracking with particles however cannot leave aside equally accurate online image guidance. The direct translation of motion monitoring techniques from conventional X-ray therapy can provide geometrical information, but the resulting density changes in the treatment field aren’t directly modelled by the real time knowledge of the target position, a crucial aspect for proton therapy applications. Ideally, proton radiography images can provide a direct measurement of the relative stopping power changes induced by the breathing motion, and as recently shown by Palaniappan et al.^48^, a limited number of projection are enough to warp (actualise) the planning CT image. Its technical realisation for time resolved 4D imaging is however not reality yet and therefore alternative approaches have to be considered. Near(er) future applications therefore entails the use of more conventional in room volumetric imaging technologies such as 4D cone beam CT^49^, magnetic resonance^17^ or ultrasound^50,51^, as surrogates to update the treatment plan based on the patient’s breathing and synchronise the delivery.

## Methods

Our approach starts off with conventional 3D treatment plan optimisation on a reference stationary image taken from the available set of 4D CT phases or a meaningful combination of these^52^. All pencil beams are then tracked over the 4D images deriving the required lateral and energy corrections that are required to restore the Bragg peaks positions in the deforming patient anatomy. This results in a pool of treatment plans, one per breathing phase, which is further processed by sorting each by energy, to generate a spot list for tumour tracking that takes into account the dynamic of the breathing and dose delivery.

### Spot tracking over 4D images

**Figure 4:**
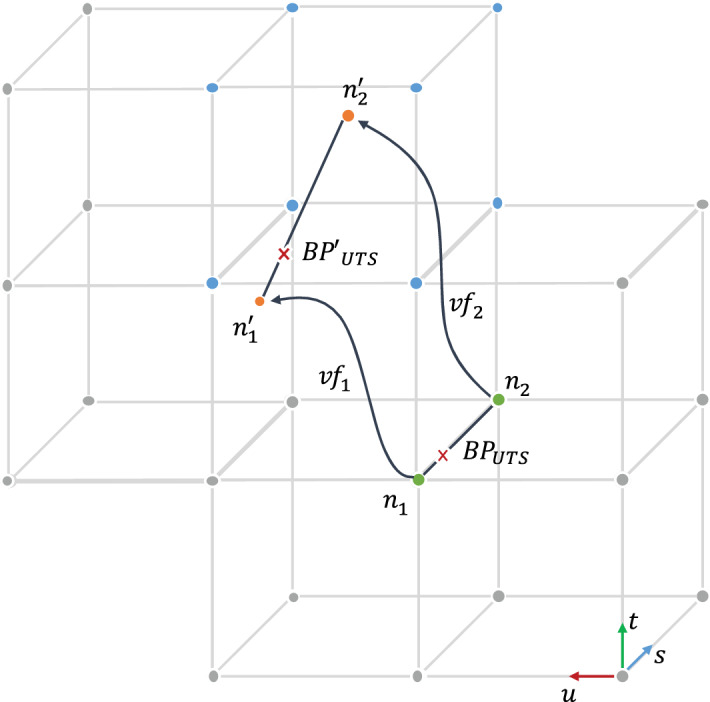
Tracking the Bragg peak position over 4D images. Representation in treatment field $\left(\mathrm{uts}\right)-\mathrm{coordinates}$.

The reference treatment plan contains the list of pencil beams whose position is given by the scan spot transversal coordinates $(u,t)$ as projected onto the machine isocentric plane in gantry coordinates and the respective nominal beam energy $(E)$. It is however convenient for our purposes to give a geometrical representation of spots with respect to isocenter by defining a third dimension $(s)$, in depth, along the beam axis. With reference to Fig. 4, $\left(uts\right)-coordinates$ define a regular grid, centred at isocenter, which can be conveniently mapped onto the DICOM imaging system reference to derive the tissue density of each node from CT intensity values. Consequently, the equivalent range in water at 80% of Bragg peak fall-off $(BP)$ is calculated for each pencil beam by accumulating the tissue stopping power along the s-nodes on the beam track. Due to grid discretisation, linear interpolation among the two ${n}_{1}$,${n}_{2}$ adjacent nodes either side of the nominal water equivalent range of the spot is applied to obtain the actual ${BP}_{UTS}$ position for each given beam energy.

The deformation of the $\left(uts\right)-coordinates$ grid at each breathing phase is computed with deformable image registration between the planning image and all other phases of the 4D dataset. The resulting deformation fields are used to warp the $\overline{{n_{1} n_{2} }}$ segment (the line joining n_1_ and n_2_) to which the spot belongs, so as to obtain the positions of its endpoints (${n}_{1}^{{\prime}},{n}_{2}{^{\prime}}$), in each breathing phase. In a simplified approach, the Bragg peaks of the spots in the nominal plan are expressed as relative distances from *n*_1_ along $\overline{{n_{1} n_{2} }}$ and the updated ${BP{^{\prime}}_{UTS}}$ are located along $\stackrel{-}{{n}_{1}^{{\prime}}{n}_{2}^{{\prime}}}$ at a proportional distance from ${n}_{1}^{{\prime}}$, preserving its relative position along the new segment. Finally, the actual water equivalent range, and so the energy, of each warped spot position is calculated by trilinear interpolation of the eight vertices defining the bounding box of ${BP{^{\prime}}_{UTS}}$ (blue nodes in Fig. 4).

As a result of this process, the reference plan is replicated for each available breathing phase, making sure that the Bragg peak positions are tracked to the corresponding anatomical location as in the reference treatment plan.

### Spot sequence optimisation

Spot sorting is based around three concepts referred to as energy bands, energy sequencing and beam pauses.

#### Energy bands

The design and configuration of the magnets along the beamline determine its momentum acceptance. As such, the term “energy band” refers to the range of energies around the nominal momentum of particles that can be transported through the beamline for each given beamline tune setting. In most existing proton gantries, this gap is small and limited to just a few percent of the nominal energy. However, it is possible to generate high-resolution beamline tunes so that the corresponding energy bands are adjacent and cover the full energy range required in a treatment plan.

#### Energy sequencing

**Figure 5:**
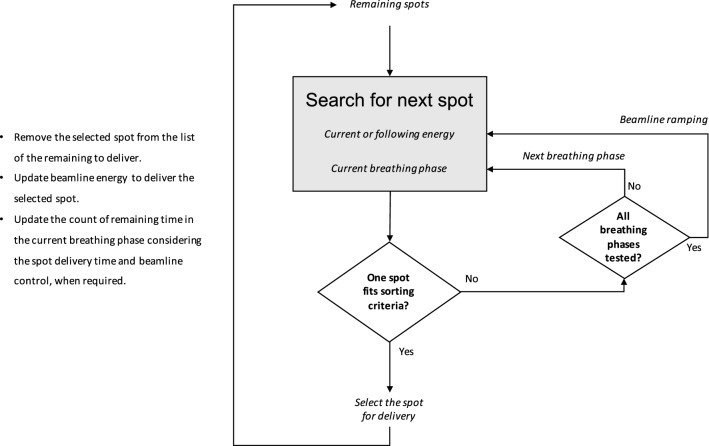
Flow chart of the energy sequencing procedure for tumour tracking spot list generation.

Due to technical constraints, magnetic ramping of the beamline is typically performed only in one direction and consequently, random jumps between energy bands should be avoided. Leveraging the high flexibility of PBS units in controlling the lateral beam position, a new scan path for tumour tracking can be generated, prioritising energy sorting to avoid meandering changes. Following the standard high-to-low ramping scheme, the spot list from the nominal treatment plan, together with its replicas for each respiratory phase are globally sorted by decreasing energy in order to identify the one that, including tracking offsets, has the highest energy. Such spots, and their corresponding breathing phase, are the starting point to create a plausible sequence to be delivered synchronised to the patient motion. Starting from this initial condition, spots in descending order of energy are progressively selected for delivery on the respiratory phases considering the time needed for dose delivery, beamline tune changes and the patient’s breathing period. Once a spot is selected for delivery, it is removed from the pool of all phases, thus preserving the number of spots and total dose of the nominal treatment plan. In such a way, the correct descending progression of energy tunes is observed during treatment, while tracking the tumour motion. The workflow of this procedure is shown in Fig. 5.

#### Beam pauses

In this implementation of tumour tracking, monitoring breathing motion is essential to trigger the start of treatment and keep the synchrony between the energy sequence and respiratory phases. However, due to the interplay between the energy sequencing and patient motion, certain phases may be empty or, more likely, the number of spots in sorted energy order may not be enough to fill the duration of each breathing phase. In such cases, the delivery has to be paused to keep the synchrony and preserve the treatment structure.

**Figure 6:**
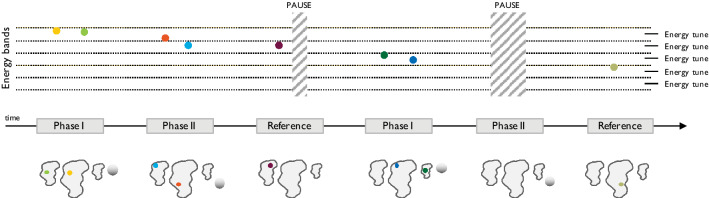
Motion correlated delivery of a simple tracking plan from Fig. 1 making use of the energy bands concept to implement tumour tracking energy offsets.

A schematic of spot sorting is shown in Fig. 6 for the simplified tracking plan of Fig. 1 in the introduction section. In this example, the spot with the highest energy, including tracking offset, belongs to the first breathing phase (Phase I, yellow spot). Three spots can be delivered before switching to the second energy band in the breathing Phase II. When coming to the next breathing phase (Reference), no spots are left to be delivered in the current or immediately following energy band and therefore beam is paused until the end of Reference phase, thus ensuring continuity in the progression of energies. Afterwards the treatment proceeds by moving to the following energy band and again a full pause on Phase II is required before completing the treatment with the last spot.

### Advanced energy meandering and rescanning

**Figure 7:**
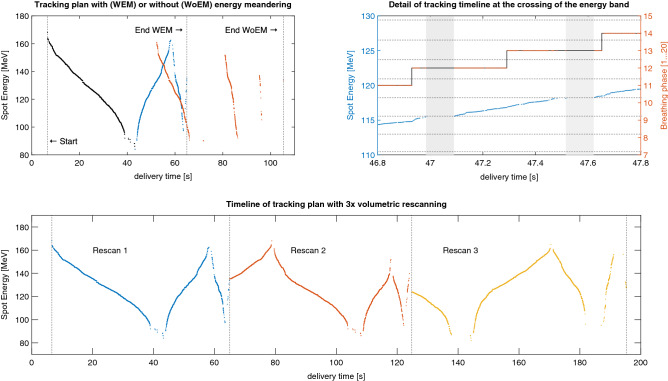
simulated delivery of tracking treatment plans for a liver cancer patient (P1) considering $$  {\raise0.7ex\hbox{${\Delta p}$} \!\mathord{\left/ {\vphantom {{\Delta p} p}}\right.\kern-\nulldelimiterspace} \!\lower0.7ex\hbox{$p$}} =  \pm 0.6\%    $$ momentum acceptance. Top-left panel: treatment time comparison between tracking deliveries with (WEM) and without (WoEM) energy meandering. The two deliveries have in common the first energy ramp down (plotted in black) and differ afterwards, WEM (blue) almost halving the treatment duration of WoEM (red). Lower panel: tracking with three volumetric rescans and energy meandering. A 1-s detail at time 46.8 s is given in the top-right panel showing the interplay between the delivery (left y-axis) and the progression of breathing phases (right y-axis). Energy bands are plotted as dotted horizontal lines and two pauses due to the beamline tune changes between subsequent bands are displayed as grey areas.

Since it is likely that the complete treatment plan cannot be delivered in a single beamline ramp down, the dead time in this type of tumour tracking treatments could be substantial due to the need to ramp through the energies multiple times. The impact of that on the treatment efficiency is shown in Fig. 7 (top-left panel—orange data) for the liver case P1 considering 9.3 s ramping time (a figure representative of PSI Gantry 2). We introduce therefore energy meandering^20^ such that when the lowest tune is reached, the energy progression is inverted and remaining spots delivered in ascending energy order. This process, repeatedly applied until treatment completion, ensures minimal dead time, bringing tracking treatments virtually close to 100% duty cycle (Fig. 7, top—left panel—blue data).

Volumetric rescanning can also be applied in this context, with only a slight modification of the energy sequencing routine. Following conventional methods^42^, the nominal dose of each pencil beam is divided by the rescan factor to obtain actual weights per-scan. After the first scan through the whole treatment field, the first spot of the next rescan is selected from those available in the current energy band of the beamline, and breathing phase that the patient is in. It is therefore most probable that pencil beams are rescanned to different breathing phases with specific tracking offsets, thus preserving the stochasticity of the method that makes rescanning effective. No methodological constraints limit its combination with energy meandering, making possible efficient tumour tracking with rescanning Fig. 7 (lower panel).

## Data Availability

The data presented in this study are available from the senior author (S.S.) or first author (G.F.) upon reasonable request.

## References

[1] Bert C, Durante M (2011). Motion in radiotherapy: Particle therapy. Phys. Med. Biol..

[2] Knopf A-C (2016). Required transition from research to clinical application: Report on the 4D treatment planning workshops 2014 and 2015. Phys. Med..

[3] Riboldi M, Orecchia R, Baroni G (2012). Real-time tumour tracking in particle therapy: Technological developments and future perspectives. Lancet Oncol..

[4] Shimizu S (2014). A proton beam therapy system dedicated to spot-scanning increases accuracy with moving tumors by real-time imaging and gating and reduces equipment size. PLoS ONE.

[5] Fattori G (2014). Commissioning of an integrated platform for time-resolved treatment delivery in scanned ion beam therapy by means of optical motion monitoring. Technol. Cancer Res. Treat..

[6] Low, D. A. in *Advances in Radiation Oncology* **60,** 41–67 (Springer International Publishing, 2017).

[7] Oborn BM (2017). Future of medical physics: Real-time MRI-guided proton therapy. Med. Phys..

[8] Keall PJ, Kini VR, Vedam SS, Mohan R (2001). Motion adaptive x-ray therapy: A feasibility study. Phys. Med. Biol..

[9] Schweikard A, Glosser G, Bodduluri M, Murphy MJ, Adler JR (2010). Robotic motion compensation for respiratory movement during radiosurgery. Comput. Aided Surg..

[10] Kamino Y (2006). Development of a four-dimensional image-guided radiotherapy system with a gimbaled X-ray head. Int. J. Radiat. Oncol. Biol. Phys..

[11] Buzurovic I, Huang K, Yu Y, Podder TK (2011). A robotic approach to 4D real-time tumor tracking for radiotherapy. Phys. Med. Biol..

[12] Lang S (2014). Development and evaluation of a prototype tracking system using the treatment couch. Med. Phys..

[13] Depuydt T (2011). Geometric accuracy of a novel gimbals based radiation therapy tumor tracking system. Radiother. Oncol..

[14] Sawant A (2008). Management of three-dimensional intrafraction motion through real-time DMLC tracking. Med. Phys..

[15] Hansen R (2016). Electromagnetic guided couch and multileaf collimator tracking on a TrueBeam accelerator. Med. Phys..

[16] Ehrbar S (2017). Comparison of multi-leaf collimator tracking and treatment-couch tracking during stereotactic body radiation therapy of prostate cancer. Radiother. Oncol..

[17] Kurz C (2020). Medical physics challenges in clinical MR-guided radiotherapy. Radiat. Oncol..

[18] Zhang Y, Knopf A, Tanner C, Lomax AJ (2014). Online image guided tumour tracking with scanned proton beams: A comprehensive simulation study. Phys. Med. Biol..

[19] Lüchtenborg R, Saito N, Durante M, Bert C (2011). Experimental verification of a real-time compensation functionality for dose changes due to target motion in scanned particle therapy. Med. Phys..

[20] Actis O, Mayor A, Meer D, Weber DC (2018). Precise beam delivery for proton therapy with dynamic energy modulation. J. Phys. Conf. Ser..

[21] Haberer T, Becher W, Schardt D, Kraft G (1993). Magnetic scanning system for heavy ion therapy. Nucl. Inst. Methods Phys. Res. A.

[22] Grözinger SO, Li Q, Rietzel E, Haberer T, Kraft G (2004). 3D online compensation of target motion with scanned particle beam. Radiother. Oncol..

[23] Bert C (2010). Dosimetric precision of an ion beam tracking system. Radiat. Oncol..

[24] Saito N (2009). Speed and accuracy of a beam tracking system for treatment of moving targets with scanned ion beams. Phys. Med. Biol..

[25] Wan W (2015). Alternating-gradient canted cosine theta superconducting magnets for future compact proton gantries. Phys. Rev. Spec. Topics Accel. Beams.

[26] Gerbershagen A, Meer D, Schippers JM, Seidel M (2016). A novel beam optics concept in a particle therapy gantry utilizing the advantages of superconducting magnets. Z. für Med. Phys..

[27] Gerbershagen A, Calzolaio C, Meer D, Sanfilippo S, Schippers M (2016). The advantages and challenges of superconducting magnets in particle therapy. Supercond. Sci. Technol..

[28] Schippers, M., Meer, D. & Gerbershagen, A. Particle therapy gantry with an energy degrader and an achromatic final bending system—European Patent Office—EP 3167933 A1. 1–14 (2017).

[29] Bert C, Rietzel E (2007). 4D treatment planning for scanned ion beams. Radiat. Oncol..

[30] van de Water S, Kreuger R, Zenklusen S, Hug E, Lomax AJ (2009). Tumour tracking with scanned proton beams: Assessing the accuracy and practicalities. Phys. Med. Biol..

[31] Zhang Y, Huth I, Wegner M, Weber DC, Lomax AJ (2016). An evaluation of rescanning technique for liver tumour treatments using a commercial PBS proton therapy system. Radiother. Oncol..

[32] Boye D, Lomax T, Knopf A (2013). Mapping motion from 4D-MRI to 3D-CT for use in 4D dose calculations: A technical feasibility study. Med. Phys..

[33] Shackleford JA, Kandasamy N, Sharp GC (2010). On developing B-spline registration algorithms for multi-core processors. Phys. Med. Biol..

[34] Schippers JM (2006). The use of protons in cancer therapy at PSI and related instrumentation. J. Phys..

[35] Klimpki G (2018). The impact of pencil beam scanning techniques on the effectiveness and efficiency of rescanning moving targets. Phys. Med. Biol..

[36] Chaudhri N (2010). Ion-optical studies for a range adaptation method in ion beam therapy using a static wedge degrader combined with magnetic beam deflection. Phys. Med. Biol..

[37] Weber U, Becher W, Kraft G (2000). Depth scanning for a conformal ion beam treatment of deep seated tumours. Phys. Med. Biol..

[38] Pedroni E (1995). The 200-MeV proton therapy project at the Paul Scherrer Institute: Conceptual design and practical realization. Med. Phys..

[39] Titt U (2010). Adjustment of the lateral and longitudinal size of scanned proton beam spots using a pre-absorber to optimize penumbrae and delivery efficiency. Phys. Med. Biol..

[40] Michiels S (2018). Patient-specific bolus for range shifter air gap reduction in intensity-modulated proton therapy of head-and-neck cancer studied with Monte Carlo based plan optimization. Radiother. Oncol..

[41] Pedroni E (2005). Experimental characterization and physical modelling of the dose distribution of scanned proton pencil beams. Phys. Med. Biol..

[42] Zenklusen SM, Pedroni E, Meer D (2010). A study on repainting strategies for treating moderately moving targets with proton pencil beam scanning at the new gantry 2 at PSI. Phys. Med. Biol..

[43] Bernatowicz K, Lomax AJ, Knopf A (2013). Comparative study of layered and volumetric rescanning for different scanning speeds of proton beam in liver patients. Phys. Med. Biol..

[44] Bula C, Belosi MF, Eichin M, Hrbacek J, Meer D (2019). Dynamic beam current control for improved dose accuracy in PBS proton therapy. Phys. Med. Biol..

[45] Eley JG, Newhauser WD, Lüchtenborg R, Graeff C, Bert C (2014). 4D optimization of scanned ion beam tracking therapy for moving tumors. Phys. Med. Biol..

[46] Graeff C (2014). Motion mitigation in scanned ion beam therapy through 4D-optimization. Phys. Med..

[47] Wolf ME, Anderle K, Durante M, Graeff C (2020). Robust treatment planning with 4D intensity modulated carbon ion therapy for multiple targets in stage IV non-small cell lung cancer. Phys. Med. Biol..

[48] Palaniappan P (2020). Deformable image registration of the treatment planning CT with proton radiographies in perspective of adaptive proton therapy. Phys. Med. Biol..

[49] Landry G, Hua CH (2018). Current state and future applications of radiological image guidance for particle therapy. Med. Phys..

[50] Hsu A, Miller NR, Evans PM, Bamber JC, Webb S (2005). Feasibility of using ultrasound for real-time tracking during radiotherapy. Med. Phys..

[51] Giger AT (2020). Liver-ultrasound based motion modelling to estimate 4D dose distributions for lung tumours in scanned proton therapy. Phys. Med. Biol..

[52] Wolthaus JWH, Sonke JJ, van Herk M, Damen EMF (2008). Reconstruction of a time-averaged midposition CT scan for radiotherapy planning of lung cancer patients using deformable registrationa. Med. Phys..

